# Whole-Genome Resequencing Reveals Population Structure and Loci Associated with Growth and Meat-Quality Traits in Meigu Yanying Chickens

**DOI:** 10.3390/ani16040540

**Published:** 2026-02-09

**Authors:** Yaodong Hu, Binjian Cai, Tianyu Li, Shi Tang, Silu Wang, Caiyun Sun, Binlong Chen, Peng Ren

**Affiliations:** 1 College of Animal Science, Xichang University, Xichang 615000, China; 2 College of Life Sciences and Agri-Forestry, Southwest University of Science and Technology, Mianyang 621010, China

**Keywords:** population structure, leg muscle, liver, protein content, cholesterol

## Abstract

Liangshan Meigu Yanying chicken is an indigenous high-altitude breed in Sichuan, China, but its population genetic structure and the genetic basis of key growth and meat-quality traits remain unclear. In this study, 211 Meigu Yanying chickens were whole-genome resequenced, and leg muscle weight, liver weight, leg muscle total protein content and leg muscle total cholesterol content were recorded at slaughter. After quality control, high-confidence single-nucleotide polymorphisms were used to analyze linkage disequilibrium, population structure and genome-wide associations. Linkage disequilibrium decayed rapidly with distance, and multivariate analyses indicated an overall homogeneous genetic background with only mild substructure and few closely related individuals. Genome-wide association studies for the four traits detected several significant or suggestive regions harboring biologically plausible candidate genes, including CPNE4, PLXNB2, SMAP1, SDHAF4, FAM135A, LCP1, MAPK15 and SCRIB. Gene Ontology enrichment showed that candidate genes for leg muscle weight and liver weight were mainly involved in cellular processes and tissue development, whereas those for leg muscle total cholesterol content were enriched in phosphorus metabolism and phosphate-containing compound metabolism. These results provide a genome-wide resource for Meigu Yanying chickens and lay a basis for conservation and genomic breeding aimed at improved growth performance, meat quality and nutritional value.

## Simple Summary

The Meigu Yanying chicken is an important local meat breed from the mountains of southwest China, but little is known about the genes that make it strong, productive and good to eat. In this study, we combined detailed body and meat measurements with modern genetic sequencing in 211 birds to explore how differences in their genetic code relate to growth and meat quality. We focused on leg muscle weight, liver weight, protein content in leg meat and cholesterol content in leg meat, which are all closely linked to both farmer profit and consumer health. Our analyses showed that this breed still has rich genetic diversity and only low levels of inbreeding, which is positive for long-term conservation and breeding. We also found several regions in the genetic code that were strongly associated with heavier muscles and livers, higher protein and differences in cholesterol level. These results provide a scientific basis to protect this local breed and to design breeding programs that produce chickens with better growth, tastier meat and potentially healthier nutritional profiles for consumers.

## 1. Introduction

Chicken meat, as a representative source of white meat, is playing an increasingly important role in the global consumption of animal-derived foods. Because its fat, cholesterol and iron contents are generally lower than those of red meat, chicken meat is widely regarded as more suitable for modern consumers who seek high-protein and low-fat foods, and its consumption has continued to increase over the past decades in both developed and developing countries [1,2]. In China, small- and medium-scale free-range systems based on indigenous chicken breeds not only supply high-quality animal protein, but also contribute to the maintenance of genetic diversity in poultry. Liangshan Meigu Yanying chicken is a local breed specific to the high-altitude mountainous areas of the Liangshan Yi Autonomous Prefecture in Sichuan Province. It is characterized by its large body size, strong and coarse bones, good resistance to cold and disease, and good adaptability to woodland and hillside grazing, and thus represents an important genetic resource for high-quality meat production in this region [3].

Meat quality is an important breeding objective in poultry. Multiple biochemical components contribute to sensory and nutritional attributes of meat, including intramuscular fat as well as muscle protein and cholesterol. In the present study, we focus on muscle protein content and cholesterol level, which are directly relevant to nutritional value and consumer health perception. Protein content reflects the proportion of lean tissue and the capacity to supply amino acids, whereas muscle cholesterol level is closely related to cardiovascular risk and consumer perception of healthy meat [4]. Moreover, feeding regime, diet composition and chicken breed type can significantly affect lipid- and cholesterol-related traits in meat, highlighting the value of investigating their genetic basis for breeding applications [5,6,7].

For poultry breeds with high reproductive rates, long-term closed selection within a limited population can easily lead to increased inbreeding coefficients, resulting in inbreeding depression, such as reduced fertility and hatchability and deterioration in growth and reproductive traits [8,9]. Therefore, for local chicken breeds under systematic selection, maintaining a sufficient effective population size and genetic diversity on the one hand, and controlling inbreeding and hidden relatedness through rational breeding schemes on the other hand, are key prerequisites for achieving both conservation and improvement goals. With the rapid decline in sequencing costs, population genetic analyses and genome-wide association studies (GWASs) based on high-density single-nucleotide polymorphism (SNP) chips or whole-genome resequencing have been widely used to dissect complex quantitative traits in chickens, including growth performance, carcass traits and fat deposition [10,11,12]. Many studies have shown that using dense marker information to infer population structure and build genomic relationship matrices, combined with linear mixed models for GWASs, can greatly improve mapping accuracy and reduce false positives, thus providing strong support for candidate gene identification and molecular breeding [13,14,15].

At present, studies on Meigu Yanying chicken mainly focus on external characteristics and production performance, while its population structure, genome-wide variation landscape and the genetic basis of economically relevant slaughter and nutritional traits have not been systematically investigated. To systematically address these genomic questions, modern high-fidelity (HiFi) sequencing technologies, which offer unparalleled accuracy in generating long reads and provide a powerful tool for precise variant detection and haplotype-resolved analysis [16,17]. Motivated by these advances in genomic technologies and resources, we prioritized four traits with clear breeding significance: leg muscle weight (LMW) as an indicator of carcass yield; liver weight (LW) reflecting metabolic organ development; leg muscle total protein (LMTP); and total cholesterol (LMTC), representing key nutritional and health-related attributes of edible meat. Therefore, in this study, 211 Meigu Yanying chickens were used as experimental animals. Based on whole-genome resequencing data, we characterized the distribution of SNPs across the genome and the pattern of linkage disequilibrium decay, analyzed population structure and relatedness, and performed GWAS and Gene Ontology (GO) enrichment analyses for LMW, LW, LMTP and LMTC. The objectives of this study were: (i) to describe the genomic diversity and population structure of Meigu Yanying chickens; (ii) to identify major associated regions and candidate genes for the above growth and meat-quality traits; and (iii) to explore key biological pathways related to muscle protein deposition and cholesterol metabolism. The results will provide a theoretical basis and candidate markers for conservation of this genetic resource and for genomic selection aimed at high-quality and healthier local chicken products.

## 2. Materials and Methods

### 2.1. Experimental Animals

All research animal care procedures were approved by the Institutional Animal Care and Ethics Committee of Xichang University (Permit No.: XCC2026006, approval date: 5 January 2026). A total of 211 Meigu Yanying chickens were used for whole-genome resequencing and quantitative trait analyses. The cohort comprised individuals of both sexes selected in a mixed-sex manner without sex-based stratification; therefore, sex was not included as a covariate in the GWAS models. All chickens were obtained from the local conservation and breeding system in Meigu County, including the county-level Meigu Yanying chicken breeding farm and its cooperative smallholder flocks. They were raised in the same region under local routine feeding and vaccination programs, and thus represent the main genetic background of Meigu Yanying chickens under current production conditions.

All chickens were slaughtered under standard commercial conditions, and sampling was carried out in a uniform manner. Because birds were sourced from the local conservation and breeding system, individual hatch- date records were not available for all animals; however, all individuals were sampled at the typical market stage under local production conditions. Live body weight at slaughter was recorded for each bird (mean ± SD: 3613.25 ± 521.80 g; range: 2308.98–5501.55 g). LMW and LW were measured using an analytical balance. LMTP was quantified in leg muscle extracts by the bicinchoninic acid (BCA) assay (Smith et al., 1985) and expressed as protein concentration (g/L) [18]. LMTC was determined using an enzymatic cholesterol esterase/cholesterol oxidase–peroxidase method (Allain et al., 1974) and expressed as cholesterol normalized to total protein (mmol/g protein) [19]. Biochemical measurements were performed in technical duplicate, and the mean value was used for downstream analyses; intra-assay variability was evaluated using the coefficient of variation (CV) calculated from duplicates. Tissue samples were snap-frozen in liquid nitrogen immediately after collection and then stored at −80 °C until genomic DNA extraction and subsequent analyses.

### 2.2. Data Filtering and Read Mapping to the Reference Genome

Whole-genome resequencing was performed on the DNBSEQ-T7 platform (paired-end 150 bp). For downstream analyses, raw reads were first quality-controlled and preprocessed with fastp [20] (v0.24.0), including removal of adapter sequences, trimming of low-quality bases at the read ends and filtering out reads containing excessive ambiguous bases (N), in order to ensure reliable alignment and variant calling. The high-quality reads were then aligned to the chicken reference genome GRCg7b (GCF_016699485.2_bGalGal1.mat.broiler) using the BWA-MEM algorithm [21] (v0.7.18) to generate sorted BAM files. Samtools [22] (v1.21) was used for file format conversion and index construction. PCR duplicates were marked and removed with the MarkDuplicates module of Picard (v2.18.7, https://broadinstitute.github.io/picard/ (accessed on 3 December 2025)), to further reduce false-positive variants caused by amplification bias. After alignment and duplicate removal, the mean genome-wide sequencing depth was 12.30× per individual, and the resulting ~12× whole-genome resequencing data were used for downstream population genetic analyses and GWAS.

### 2.3. SNP Calling, Genotype Quality Control and Imputation

Based on the deduplicated BAM files, SNP calling was carried out with GATK [23] (v4.6.1.0). HaplotypeCaller was run separately for each chicken to generate gVCF files, which were then merged using CombineGVCFs and jointly genotyped with GenotypeGVCFs to obtain a population-level raw variant set. To ensure robust GWAS results, multiple rounds of quality control were performed on both SNPs and individuals in PLINK [24] (v1.9.0). Individuals with a genotype missing rate > 5% were removed (--mind 0.05), SNPs with a call rate < 95% were excluded (--geno 0.05), and loci with minor allele frequency (MAF) < 0.05 (--maf 0.05) or deviating from Hardy–Weinberg equilibrium at *p* < 1 × 10^−6^ (--hwe 1 × 10^−6^ ) were filtered out at the population level. After obtaining a high-quality SNP set, missing genotypes were phased and imputed with Beagle [25] (v5.4) to improve marker completeness and the accuracy of effective allele frequency estimation. In addition, for PCA, ADMIXTURE and relatedness analyses, a set of approximately independent markers was derived from the high-quality SNPs by LD pruning using the command --indep-pairwise 50 5 0.1 in PLINK.

### 2.4. Functional Annotation of Candidate SNPs and GO Enrichment Analysis

On the basis of significant or suggestive SNPs detected in the GWAS for each trait, lead SNPs and their flanking regions (±100 kb) were defined as candidate associated intervals. All SNPs within these intervals were extracted, and regional VCF files were generated using PLINK for subsequent annotation. Gene Ontology (GO) enrichment analysis was performed in R [26] (v4.4.3) using clusterProfiler (gseGO, GSEA). GO analysis was conducted for the Biological Process (BP) category. GO annotations for *Gallus gallus* were obtained from the Bioconductor annotation packages org.Gg.eg.db (gene-to-GO mapping) and GO.db (ontology terms). The Benjamini–Hochberg procedure was used to control the false discovery rate at FDR < 0.05 for multiple testing.

To facilitate functional interpretation and prioritization of candidate genes, genes located within lead SNP ± 100 kb intervals were classified based on snpEff annotation. For each gene, all variants within the corresponding interval were ranked according to their GWAS *p* values, and the smallest *p* value was recorded as *p*_min for that gene. At the same time, according to the ANN_impact category in snpEff, genes were grouped into three tiers: Tier 1 (high-impact variants), Tier 2 (moderate-impact variants) and Tier 3 (low-impact or regulatory variants).

### 2.5. Statistical Analyses and Visualization

For population genetic analyses, PLINK was used to calculate the genotype correlation matrix and identity-by-descent (IBD) statistics. ADMIXTURE [27] (v1.3.0) was applied with K values from 2 to 8, and the number of ancestral clusters with the smallest cross-validation error was taken as the optimal K. The corresponding membership coefficients were plotted as bar plots to show the population composition of the 211 Meigu Yanying chickens. PCA was used to examine the distribution of individuals along the leading principal components, in order to assist interpretation of the ADMIXTURE results and to assess potential population stratification [28]. Linkage disequilibrium decay was analyzed using PopLDdecay [29] (v3.43). Pairwise r^2^ values were calculated for SNPs at different physical distances, and LD decay curves for the whole population and different subgroups were plotted in R to evaluate LD block size and historical recombination levels.

GWAS was performed with GEMMA [30] (v0.98.3) using a linear mixed model, fitting the first five principal components and source flock/farm (breeding batch) as fixed covariates. A Bonferroni-adjusted threshold of *p* < 0.05/M (where M is the number of approximately independent SNPs after LD pruning) was used to define genome-wide significant associations, and *p* < 1/M was used as a suggestive threshold. False discovery rate values were also calculated to aid in the selection of robust signals. Manhattan plots and Q–Q plots were generated in R using CMplot (v4.5.1) based on the GWAS output from GEMMA. All other graphical visualizations were also produced in R.

## 3. Results

### 3.1. Phenotypic Data

At slaughter age, four traits were measured in the 211 Meigu Yanying chickens: LMW, LW, LMTP and LMTC. LMW ranged from 104.74 to 421.47 g, with a mean of 244.49 g and a coefficient of variation of 26.75%, indicating substantial individual variation for this trait within the population. LW, LMTP and LMTC also showed moderate to high phenotypic variation (Table 1), providing a solid phenotypic basis for subsequent genome-wide association analyses.

### 3.2. Genome-Wide SNP Detection, Functional Annotation and Linkage Disequilibrium

Whole-genome resequencing of all samples generated about 2.93 Tb (2929.15 Gb) of clean data. The sequencing quality was high, with Q20 values ranging from 97.60% to 99.53% and Q30 values from 93.39% to 98.30%. GC content varied between 40.81% and 42.30%. The mean genome-wide coverage depth was 12.30×. The duplicate rate was low (average 0.61%, range 0.41–1.93%), and the mapping rate was high across all chickens.

**Figure 1:**
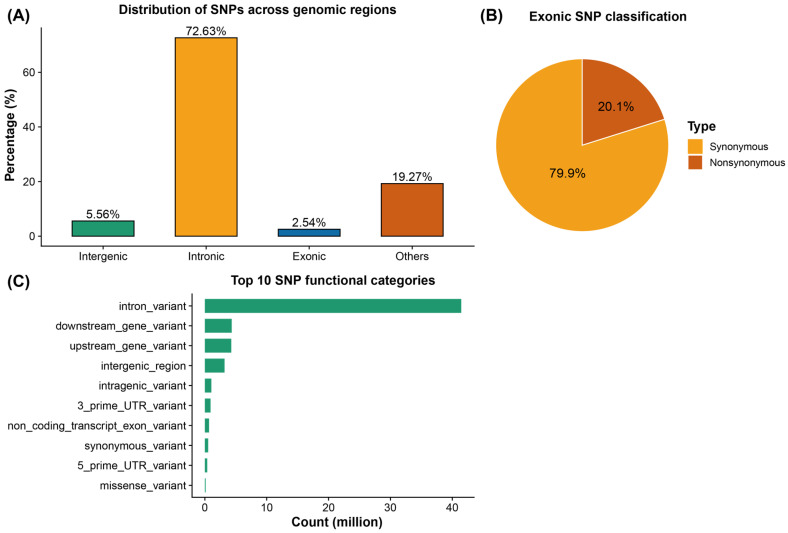
Genome-wide functional annotation of SNPs in Meigu Yanying chickens based on SnpEff. (**A**) Proportional distribution of SNPs across different genomic functional regions, including intronic, intergenic, exonic, upstream and downstream regions. (**B**) Composition of functional categories of SNPs within exonic regions, mainly including synonymous variants and missense variants. (**C**) Summary of SNP functional categories annotated by SnpEff, showing the counts of major classes such as intron_variant, upstream_gene_variant, downstream_gene_variant and intergenic_region.

**Figure 2:**
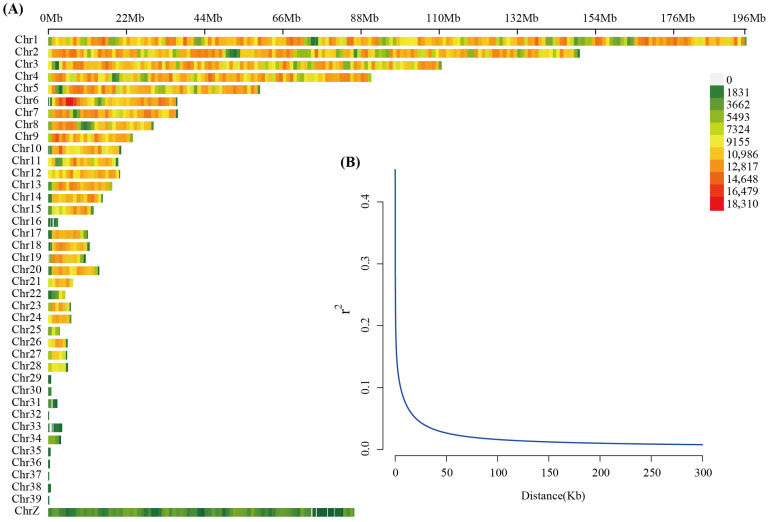
Genome-wide SNP density and linkage disequilibrium (LD) decay in Meigu Yanying chickens. (**A**) Chromosome-wise SNP density distribution based on the high-quality SNP set; color intensity indicates the number of SNPs within physical windows, illustrating the spatial distribution of variants and the uniformity of sequencing coverage along each chromosome. (**B**) LD decay curve calculated from the filtered genome-wide SNPs; the *x*-axis represents the physical distance between marker pairs (kb) and the *y*-axis the mean r^2^ value, showing the decline in LD strength with increasing physical distance.

In total, 9,945,867 high-quality SNPs were identified from the resequencing data of the 211 Meigu Yanying chickens. Functional annotation with SnpEff showed that the vast majority of variants were located in intronic regions (72.63%), with the remainder mainly distributed in intergenic regions (5.56%), exons (2.54%), and upstream, downstream and untranslated regions (Figure 1A). This pattern is consistent with previous whole-genome resequencing studies in local chickens and other livestock, in which most natural variation occurs in non-coding regions and only a small proportion of SNPs are located in coding sequences [12,31]. Within exons, 552,841 synonymous and 139,401 nonsynonymous mutations were detected, with nonsynonymous variants accounting for about 20% of exonic SNPs, similar to proportions reported in other chicken populations (Figure 1B). These nonsynonymous variants cause amino acid substitutions and represent an important resource for subsequent selection of functional candidate genes. Detailed SnpEff annotation further classified all variants into 30 functional categories, among which intron_variant, upstream_gene_variant, downstream_gene_variant and intergenic_region were the most abundant; missense_variant and splice_region_variant and other types with potentially larger functional impact were also detected, indicating the presence of numerous candidate sites that may participate in regulatory and coding processes (Figure 1C). Chromosome-wise SNP density (SNPs/Mb) across macrochromosomes, microchromosomes and ChrZ is shown in Figure 2A, which also provides an overview of genome-wide completeness of variant discovery.

To further characterize the spatial distribution of SNPs and the level of linkage disequilibrium in the Meigu Yanying chicken genome, SNP density was calculated in sliding windows along each chromosome and visualized as a heatmap in units of SNPs/Mb (Figure 2A). SNPs were generally distributed continuously along the physical positions of all autosomes. As expected, large macrochromosomes such as Chr1–Chr5 contained more variants in absolute number due to their greater physical length, whereas microchromosomes and ChrZ showed distinct SNP-density patterns (Figure 2A). Importantly, no extended variant-depleted regions were observed, supporting overall uniform and reliable variant discovery across the genome. Based on the filtered genome-wide SNPs, pairwise r^2^ values were calculated in bins of physical distance and used to draw the LD decay curve (Figure 2B). In the whole population, mean r^2^ was about 0.4 at very short distances (0–1 kb), then declined rapidly with increasing distance, dropping below 0.1 at around 15 kb and approaching a baseline of roughly 0.02 in the 200–300 kb interval. These results suggest that the extent of LD in Meigu Yanying chickens is relatively short and the overall LD level is low, implying a comparatively large effective population size and abundant historical recombination, which is favorable for improving the mapping resolution of GWAS.

### 3.3. Population Structure and Genomic Relatedness

**Figure 3:**
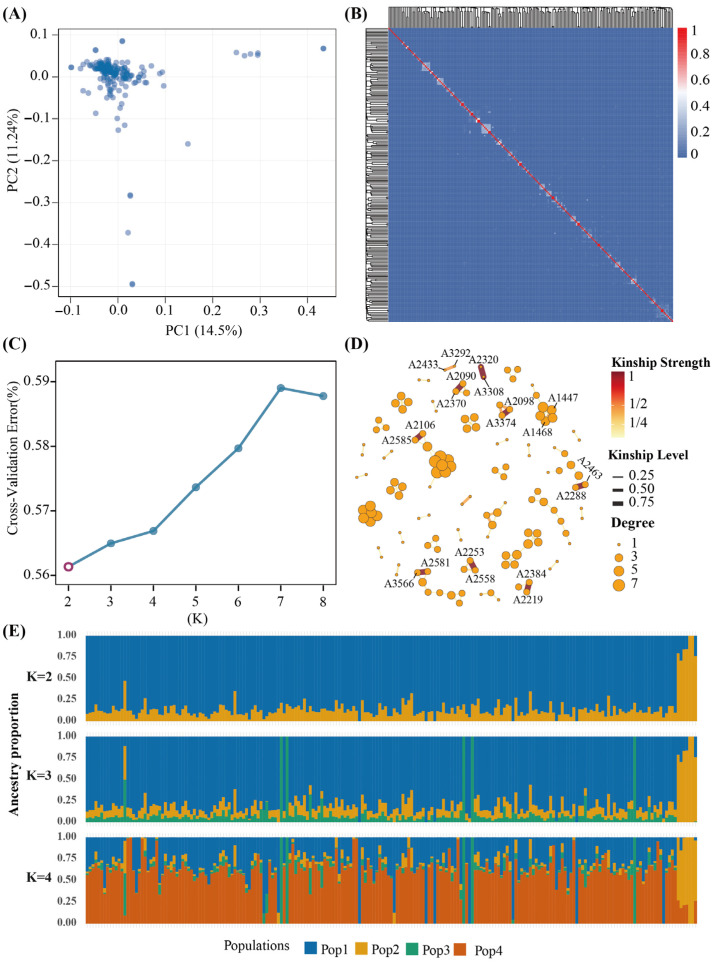
Genetic structure and kinship relationships in the Meigu Yanying chicken population. (**A**) Principal component analysis based on LD-pruned SNPs; each point represents one individual and shows the distribution of the 211 Meigu Yanying chickens along the first two principal components. (**B**) Heatmap of the pairwise kinship matrix, with color intensity indicating the strength of relatedness from low to high. (**C**) Cross-validation (CV) error curve from ADMIXTURE runs with K values ranging from 2 to 8; the circled point highlights the minimum CV error observed at K=2 (optimal K). (**D**) Force-directed kinship network constructed from pairs of individuals with PI_HAT ≥ 0.125; edge width and color reflect the magnitude of identity-by-descent (IBD), and node size corresponds to node degree. (**E**) ADMIXTURE bar plots of population structure, showing the inferred ancestry proportions of each individual at different K values (e.g., K = 2, 3, 4); different colors represent different inferred ancestral components.

Based on the LD-pruned high-quality SNP set, PCA was performed for the 211 Meigu Yanying chickens. In the PC1–PC2 space, most individuals clustered into a single main group, with only a few showing slight displacement along one of the principal components, indicating that the population is genetically relatively homogeneous overall but exhibits some degree of substructure or a continuous genetic gradient (Figure 3A). This pattern is consistent with the results of the subsequent admixture analysis.

To characterize genomic relatedness among individuals, a pairwise kinship matrix was constructed from PI_HAT values calculated in PLINK, and a clustered heatmap was drawn (Figure 3B). For the vast majority of pairs, PI_HAT values were close to zero, while only a few pairs showed clearly elevated PI_HAT, suggesting a generally low level of inbreeding in the population and the presence of only a few small family clusters with closer relationships rather than large groups of close relatives.

For population structure inference, ADMIXTURE was run with K values from 2 to 8, and the optimal K was determined according to the cross-validation (CV) error. The CV curve reached its minimum at K = 2 and then increased or fluctuated upwards with larger K values, supporting the presence of two main ancestral components in the Meigu Yanying chicken population (Figure 3C). At K = 2, the bar plot of ancestry proportions showed that most individuals were dominated by one ancestral component, whereas a small number of individuals carried mixtures of the two components in different proportions. When K was increased to 3 or 4, additional components could be extracted, but the overall pattern remained dominated by two major components, and the ancestry proportions showed a continuous distribution across individuals rather than discrete clusters strictly corresponding to sampling batches (Figure 3E).

To visualize the relatedness network more intuitively, an undirected graph was constructed using pairs of individuals with PI_HAT ≥ 0.125 as edges. After merging multiple edges, a force-directed layout was used to draw the network (Figure 3D). Edge color and width were mapped to PI_HAT values, node size reflected node degree, and nodes involved in any edge with PI_HAT ≥ 0.25 were labeled. The network contained only a few small, locally dense clusters connected by high PI_HAT edges, whereas most individuals were connected only by edges with lower PI_HAT, and no large highly related groups were observed. This is consistent with the overall pattern of weak relatedness with a few close pairs seen in the IBD heatmap.

Taken together, the PCA, IBD heatmap, admixture and relatedness network results indicate that the Meigu Yanying chickens analyzed in this study form a population with a relatively homogeneous genetic background, mild local substructure and a small number of close relatives. This pattern suggests that the population retains good genetic diversity and an adequate effective population size, and also justifies the inclusion of principal components and a genomic relationship matrix in the GWAS to account for population structure and cryptic relatedness.

### 3.4. Genome-Wide Association Analyses for the Four Traits

**Figure 4:**
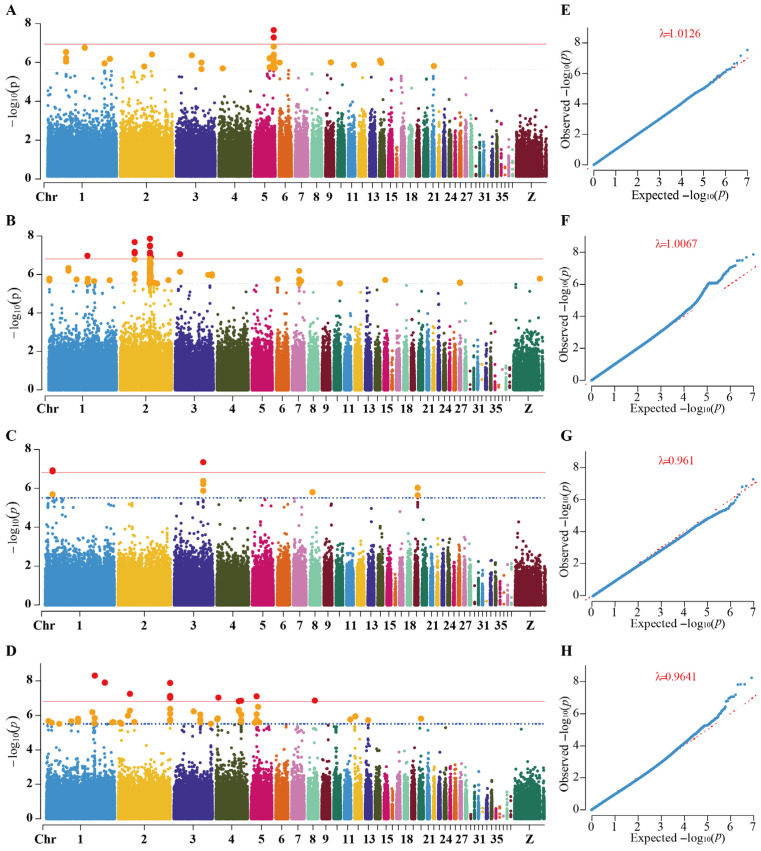
Genome-wide association results for four traits in Meigu Yanying chickens. (**A**–**D**) Manhattan plots for LMW, LW, LMTP and LMTC, respectively. The *x*-axis shows chromosomes ordered by physical position, and the *y*-axis shows –log_10_(*p*). Points are colored alternately to distinguish chromosomes. SNPs exceeding the suggestive threshold are highlighted in orange, and SNPs exceeding the genome-wide significance threshold are highlighted in red. The red horizontal line indicates the genome-wide significance threshold (*p* = 0.05/324,127), and the blue dotted horizontal line indicates the suggestive threshold (*p* = 1/324,127). Each point represents one SNP. (**E**–**H**) Q–Q plots for the corresponding traits. The *x*-axis shows the expected –log_10_(*p*), and the *y*-axis shows the observed –log_10_(*p*). The curves closely follow the diagonal line and deviate only at the tail of small *p* values, suggesting no obvious systematic inflation and good credibility of the most significant signals. Note: because of the large number of SNPs, a filtering step was applied for plotting. SNPs with *p* values between 1 × 10^−2^ and the suggestive threshold were randomly subsampled for display, and duplicate SNPs with identical *p* values were removed. Genomic inflation factor (λ) is shown in each QQ plot.

Based on the LD-pruned set of independent SNPs, linear mixed models were used to perform GWAS for LMW, LW, LMTP and LMTC, with the first five principal components and the genomic relationship matrix fitted to account for population structure and individual relatedness. Using *p* < 0.05/M (M = 324,127) as the genome-wide significance threshold and 1/M as the suggestive threshold, 57, 62, 37 and 89 independent candidate associated regions with *p* < 1 × 10^−5^ were identified for LMW, LW, LMTP and LMTC, respectively (Supplementary Table S1). Among these, 2, 12, 3 and 13 loci reached the Bonferroni-corrected genome-wide significance level for the four traits, and an additional 32, 138, 8 and 47 loci were classified as suggestive. The Q–Q plots closely followed the expected diagonal line, with deviations only at the tail of small *p* values, indicating adequate control of confounding in the models and good reliability of the most significant association signals. The genomic inflation factors (λ) were close to 1 (λ = 1.0126, 1.0067, 0.9510 and 0.9641 for panels E–H, respectively), indicating minimal test statistic inflation and effective control of population structure (Figure 4).

From the perspective of chromosomal distribution, lead SNPs for LMW were mainly located on chromosomes 1 and 5, whereas candidate loci for LW were highly enriched on chromosomes 1 and 2, suggesting that these chromosomes harbor important QTL regions affecting body growth and visceral development. The number of association signals for LMTP was relatively small, but clear peaks were observed around 20–21 Mb on the proximal part of chromosome 1 and near 82.3 Mb on chromosome 3. Lead SNPs for LMTC were more widely distributed, with a marked enrichment on chromosome 1 and multiple strong peaks also detected on chromosomes 2, 3, 4 and 5 (Table 2 and Table S1), indicating a typical polygenic architecture for leg muscle cholesterol content.

At the single-locus level, the most significant signal for LMW was located at 55.27 Mb on chromosome 5 (5_55268309_C_T, *p* = 2.89 × 10^−8^). The ±100 kb window around this SNP contained two candidate genes, one of which was classified as a Tier 1 gene, suggesting a potential direct effect on LMW. For LW, the top SNP was at chr2:86.46 Mb (2_86456028_G_A, *p* = 1.37 × 10^−8^), and an additional association peak was detected at chr2:42.10 Mb with *CPNE4* prioritized as a Tier 1 candidate, implying that membrane-related signal transduction may contribute to the regulation of liver weight. The strongest association for LMTP was at chr3:82.34 Mb (3_82338258_G_A, *p* = 4.56 × 10^−8^), where Tier 2 candidate genes such as *SMAP1*, *SDHAF4* and *FAM135A* were located; another peak was observed in the chr1:20.85–21.01 Mb interval, represented by candidates including *PLXNB2* and *ZNF800*. For LMTC, the most significant signal was mapped to chr1:139.19 Mb (1_139186656_C_T, *p* = 5.00 × 10^−9^). Strong association peaks were also found near chr1:167.91 Mb, where *LCP1* was classified as a Tier 1 candidate, and around chr2:148.90 Mb, where *MAPK15* and *SCRIB* were classified as Tier 2 candidates, suggesting that cytoskeletal remodeling, *MAPK* signaling and maintenance of cell polarity may be closely involved in leg muscle cholesterol deposition.

### 3.5. GO Enrichment Analysis of Candidate Genes

**Figure 5:**
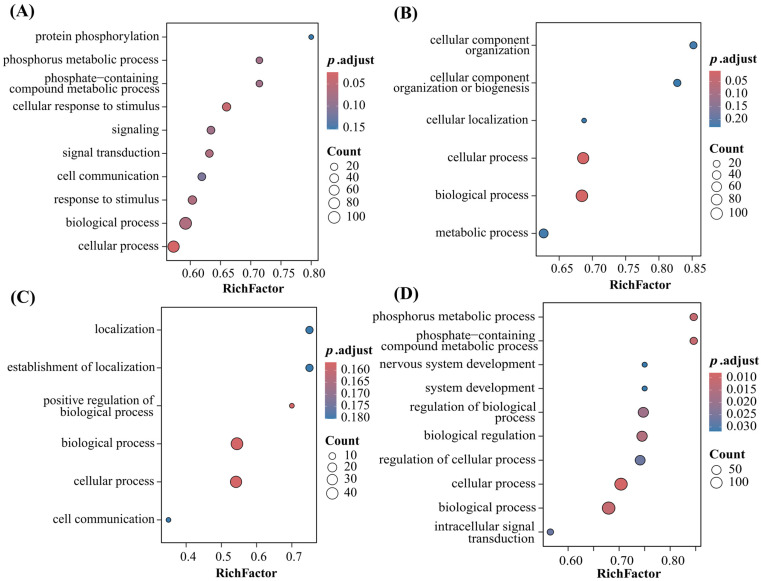
GO biological process enrichment analysis of candidate genes for four traits in Meigu Yanying chickens. (**A**–**D**) Bubble plots of GO BP enrichment for candidate genes associated with LMW, LW, LMTP and LMTC, respectively. The *x*-axis shows the RichFactor and the *y*-axis the GO term names. Bubble size represents the HitCount (number of enriched genes), and bubble color indicates the q value or adjusted *p* value. Only representative GO terms with relatively strong enrichment and clear biological relevance are shown for each trait; full enrichment results are provided in the Supplementary Table S1.

To further clarify the biological functions of genes within the candidate regions, genes classified as Tier 1–3 in the lead intervals for each trait were used as gene sets, and Gene Set Enrichment Analysis (GSEA) was performed for Gene Ontology biological process (GO BP) terms. In total, 28, 6, 6 and 73 GO BP terms were enriched for LMW, LW, LMTP and LMTC, respectively, with 2, 2, 0 and 52 terms reaching significance at q < 0.05 (Table 3 and Table S1). The enrichment profiles differed markedly among traits (Figure 5).

For LMW, candidate genes were mainly enriched in cellular process (GO:0009987, q = 0.019) and cellular response to stimulus (GO:0051716, q = 0.032) (Figure 5A), indicating that genes involved in cell growth, stress responses and signal transduction play important roles in variation in leg muscle weight. For LW, candidate genes were significantly enriched in broad basic processes such as biological_process (GO:0008150, q = 0.007) and cellular process (Figure 5B), suggesting that the genetic basis of liver weight is largely related to general biological processes including cell proliferation, tissue development and metabolic homeostasis.

In contrast, the number of enriched GO terms for LMTP was small, and no term reached significance at q < 0.05. The term with the lowest q value was positive regulation of biological process (GO:0048518, q ≈ 0.14) (Figure 5C), which overall suggests limited functional convergence among LMTP-related candidate genes, possibly due to the current sample size and relatively small effect sizes. By comparison, *LMTC* showed the strongest functional enrichment signals, with 73 GO BP terms identified, of which 52 were significantly enriched at q < 0.05 (Supplementary Table S1). Besides cellular process (GO:0009987, q = 0.004) and biological_process, *LMTC* candidate genes also showed strong enrichment in phosphorus metabolic process (GO:0006793) and phosphate-containing compound metabolic process (GO:0006796) (Figure 5D), indicating that gene networks regulating phosphorylation and turnover of phosphorus-containing metabolites may play a central role in leg muscle cholesterol deposition. This pattern is consistent with the functional characteristics of candidates such as *LCP1* and *MAPK15*, which are involved in cytoskeletal remodeling and *MAPK* signaling pathways.

## 4. Discussion

### 4.1. Genetic Diversity and Population Structure of Meigu Yanying Chickens

In this study, nearly ten million high-quality SNPs were identified in 211 Meigu Yanying chickens. The vast majority of variants were located in intronic and intergenic regions, with exonic SNPs accounting for only about 2.5%, and roughly 20% of exonic variants being missense mutations. This distribution pattern is highly consistent with resequencing results reported for various meat- and egg-type lines [12,32,33], indicating good completeness and reliability of variant detection in the present dataset. Genome-wide LD decay analysis showed that r^2^ was about 0.4 within 0–1 kb, dropped below 0.1 at around 15 kb, and approached the baseline level in the 200–300 kb interval. Overall, the extent of LD was shorter than that observed in many commercial broiler populations subjected to intensive directional selection [2,30,34,35]. These findings suggest that Meigu Yanying chickens still maintain a relatively large effective population size and abundant historical recombination, which is favorable for fine-mapping in GWAS and also indicates that this breed has not undergone long-term strong artificial selection in a closed nucleus, leaving room for further genetic improvement.

PCA and ADMIXTURE analyses showed that Meigu Yanying chickens form a relatively continuous genetic population that can be decomposed into two major ancestral components, but without clearly separated subpopulations; instead, ancestry proportions vary along a continuous gradient. The IBD heatmap and relatedness network further revealed that only a few pairs of individuals exhibited high PI_HAT values, and the overall level of inbreeding was low. This pattern is similar to recent whole-genome studies of other Chinese indigenous chicken populations, where local breeds generally retain high genetic diversity while showing some degree of regional or family-based substructure [33,36,37]. Therefore, in long-term selection and conservation of Meigu Yanying chickens, it will be important to avoid repeated mating among individuals with high IBD values to prevent rapid accumulation of inbreeding, and at the same time to maintain a balanced contribution from individuals representing different ancestral components, so as to preserve overall genetic diversity.

In the GWAS models of this study, both the first five principal components and the genomic relationship matrix were included, which effectively controlled for confounding due to population structure and cryptic relatedness. The Q–Q plots closely followed the expected diagonal, with deviations only at the extreme tail of small *p* values, indicating that the detected significant signals are generally robust. This mixed-model strategy based on high-density SNPs is consistent with approaches commonly used in GWAS for complex traits in chickens [10,11], and provides a statistical foundation for future implementation of genomic selection in this breed.

### 4.2. Genetic Architecture of Growth-Related Traits (LMW and LW)

LMW and LW are important indicators of growth and carcass traits in chickens and are closely related to carcass yield, nutrient metabolism and even drug metabolism capacity. In this study, 57 and 62 independent lead regions were identified for these two traits, with 2 and 12 loci, respectively, surpassing the stringent Bonferroni-corrected threshold, and the remaining loci showing suggestive evidence of association. This pattern is consistent with observations in other chicken populations, where body weight, muscle weight and other growth traits typically display a polygenic architecture, with each QTL having a modest effect but numerous small- to medium-effect loci across the genome jointly contributing to phenotypic variation [38,39].

In terms of chromosomal distribution, lead loci for LMW were mainly clustered on chromosomes 1 and 5, whereas candidate loci for LW were highly enriched on chromosomes 1 and 2. This agrees well with previous GWAS of body weight and carcass traits in chickens, which have repeatedly highlighted these chromosomes as harboring growth-related QTL [40], suggesting the presence of conserved regulatory regions involved in growth and organ development. In the present study, the most significant signal for LMW was located near chr5:55.27 Mb, and the top locus for LW was at chr2:86.46 Mb. Several candidate genes in these regions are involved in cytoskeletal remodeling, signal transduction and cell adhesion, and may influence leg muscle and liver weight by affecting myocyte size, muscle fiber composition, or hepatocyte proliferation and hypertrophy. Notably, within the LW-associated region at chr2:42.10 Mb, *CPNE4* was prioritized as a Tier 1 candidate gene. *CPNE4* belongs to the copine family and encodes a calcium-dependent phospholipid-binding protein that has been implicated in vesicle trafficking, membrane signaling and cytoskeletal regulation [41]. Copine proteins have been reported to play important roles in nervous system development and maintenance of synaptic morphology [42]. Although there is currently no direct evidence linking *CPNE4* to liver growth in poultry, hepatocytes are highly dependent on vesicular transport and signaling to regulate lipid export and protein synthesis. It is therefore plausible that *CPNE4* and its related pathways may influence liver mass by modulating hepatocyte membrane structure and intracellular signaling networks. This hypothesis requires further validation by transcriptomic, proteomic and functional studies.

### 4.3. Genetic Basis of Muscle Composition Traits (LMTP and LMTC)

Compared with growth traits, LMTP and LMTC are more directly related to the nutritional value and health attributes of meat. LMTP reflects the relative content of lean muscle protein, whereas LMTC is closely associated with consumer demand for low-cholesterol meat products [43]. In this study, 37 lead regions were detected for LMTP, but only a limited number of GO terms showed enrichment and none reached significance at q < 0.05; only moderate enrichment was observed for terms such as positive regulation of biological process. This likely indicates that LMTP is strongly influenced by environmental factors (e.g., diet composition, slaughter age, muscle water content), resulting in relatively small effect sizes at individual loci under the current sample size. Future work could increase sample size, improve phenotypic measurement accuracy and combine traits such as muscle fiber type and muscle moisture content to perform multi-trait GWAS or genomic structural equation modeling, in order to further dissect the genetic network underlying muscle protein deposition.

In contrast, LMTC showed much richer genetic signals. A total of 89 lead regions and 73 GO terms were obtained, among which 52 were significantly enriched at q < 0.05, indicating a relatively well-defined genetic architecture for leg muscle cholesterol content in Meigu Yanying chickens. Besides cellular process and biological process, the most prominent GO terms included phosphorus metabolic process and phosphate-containing compound metabolic process, which are related to phosphorylation and phosphorus-containing metabolites. This suggests that many candidate genes participate in phosphorus metabolism and networks involving phospholipids and phosphorylation signaling. Previous studies have shown that phospholipids, including phosphatidylinositol and other phosphorus-containing lipids, are key components of cell membranes, and their metabolic status largely determines the distribution and transport of cholesterol between membranes and lipid droplets [44]. In addition, several lipid metabolism–related signaling pathways, such as the MAPK pathway, rely heavily on tight regulation of protein phosphorylation and dephosphorylation [45]. Therefore, the significant enrichment of LMTC candidate genes in phosphorus metabolism–related GO terms is highly consistent with the central role of phosphorylation–dephosphorylation switches and phospholipid remodeling in cholesterol metabolism.

At the single-gene level, several LMTC-associated regions contained genes that have been implicated in lipid metabolism or energy homeostasis in other species. *LCP1*, located at chr1:167.91 Mb, was classified as a Tier 1 candidate. *LCP1* encodes a classical actin-binding protein originally identified in hematopoietic cells and widely involved in cytoskeletal remodeling, adhesion and cell migration [46]. Recent work in *3T3-L1* adipocytes has shown that knockdown of *LCP1* markedly affects the expression of browning/thermogenesis-related genes and lipid droplet morphology, suggesting a regulatory role in lipid and energy metabolism [47]. Combined with the present findings, it is reasonable to speculate that *LCP1* may influence the organization of the cytoskeleton and membrane structures in muscle cells, thereby modulating the distribution and mobilization of lipid droplets or cholesterol within myocytes.

Another important candidate, *MAPK15* (also known as *ERK7*/*ERK8*), is located near the association peak at chr2:148.90 Mb and was assigned to Tier 2. *MAPK15* is an atypical member of the MAPK family that participates in autophagy regulation and has been shown to exert protective effects on hepatic lipid homeostasis [48]. Colecchia et al. [49] reported that *MAPK15* modulates autophagy by interacting with ATG8 family proteins, thereby influencing the turnover of proteins and lipids within cells. More recent animal studies further suggest that *MAPK15* plays a key role in preventing metabolic dysfunction–related fatty liver disease, possibly by limiting excessive accumulation of intracellular lipid droplets and maintaining lipid balance [48]. Together with the phosphorus metabolism–focused enrichment profile for LMTC, these results support the hypothesis that *MAPK15*-related autophagy and phosphorylation networks may contribute to the regulation of cholesterol and other lipids in skeletal muscle, although the precise mechanisms still need to be verified in chicken models.

In addition, *SCRIB*, which is also located at chr2:148.90 Mb, was identified as another Tier 2 candidate for LMTC. *SCRIB* is a classical cell polarity regulator whose product is involved in establishing and maintaining apical–basal polarity in epithelial cells and can modulate ERK pathway activation and cell proliferation through interactions with lipid metabolism–related signaling molecules [50]. Given that cholesterol and phospholipids together form the structural basis of cell membranes, changes in cell polarity are often accompanied by remodeling of membrane lipid composition and microdomain architecture [51]. It is therefore plausible that *SCRIB* and its associated polarity network may influence the spatial organization of membranes and lipid droplets in muscle cells, thereby affecting the distribution and accumulation of cholesterol in muscle tissue.

### 4.4. Implications for Conservation and Breeding, and Study Limitations

From a population genetics perspective, this study shows that Meigu Yanying chickens have relatively high genomic diversity and an overall low level of inbreeding, with only a few small family clusters of closely related individuals. This provides a good basis for implementing directional selection without markedly compromising genetic diversity. In future conservation and breeding practice, the genomic relationship matrix constructed here can be combined with traditional pedigree information to optimize mating schemes, monitor trends in inbreeding, and, when necessary, introduce individuals representing different ancestral components in order to maintain or increase effective population size.

From a molecular breeding perspective, the GWAS results for LMW, LW, LMTP and LMTC revealed a set of stable lead regions and Tier 1–3 candidate genes with relatively high priority. These loci can serve as starting points for marker validation and marker-assisted selection. For example, SNPs around genes such as *LCP1*, *MAPK15*, *SCRIB* and *CPNE4* can be re-tested in larger Meigu Yanying populations and in other indigenous chicken breeds to evaluate the consistency of their effects and potential shared QTL across breeds. In breeding nucleus populations, monitoring changes in allele frequencies at these loci will help assess their response to ongoing selection schemes. As genomic selection becomes increasingly established in poultry breeding, incorporating these candidates into genomic breeding value (GEBV) models may enable simultaneous improvement of growth performance and meat quality traits such as muscle protein and cholesterol content [52,53].

This study also has several limitations. First, the sample size of 211 chickens provides reasonable power to detect QTL of moderate effect, but has limited power for small-effect or rare variants. Second, the analyses were conducted in a single population under specific management conditions, and the association signals have not yet been validated in other Meigu Yanying flocks or under different rearing systems; some loci may therefore show environmental or population specificity. Third, candidate genes were inferred mainly based on linkage disequilibrium and physical proximity to lead SNPs, without direct evidence from transcriptomic, proteomic or functional experiments, so interpretations at the molecular mechanism level should be treated with caution. Future work could be extended in at least three directions: (i) increasing sample size and incorporating multi-batch, multi-environment and multi-breed data, combined GWAS and meta-analysis to enhance the robustness of association signals; (ii) performing multi-omics integration (e.g., transcriptomics and epigenomics) in key QTL regions to refine causal genes and regulatory elements; and (iii) conducting functional validation for core candidates such as *LCP1*, *MAPK15* and *SCRIB* using in vitro cell models or gene editing in chicken embryos or live birds, thereby establishing a complete pipeline from associated loci to molecular mechanisms and finally to breeding application.

## 5. Conclusions

Using whole-genome resequencing data from Meigu Yanying chickens, this study characterized genome-wide SNP variation, linkage disequilibrium and population structure, and conducted GWAS and GO enrichment analyses for LMW, LW, LMTP and LMTC. The breed showed high genomic diversity and relatively short LD, while ADMIXTURE and IBD analyses indicated an overall homogeneous structure with low inbreeding and only a few small clusters of close relatives, providing a sound basis for simultaneous conservation and improvement. For growth-related traits, LMW and LW displayed typical polygenic architectures, with multiple association peaks on chromosomes 1, 2 and 5, suggesting conserved QTL regions for muscle and visceral development. LMTP had several associated intervals but weak functional convergence, implying a dispersed genetic basis that may require larger sample sizes to resolve. LMTC showed the clearest genetic and functional pattern, with numerous candidate genes enriched in phosphorus metabolism, phosphorylation and cellular processes; among them, LCP1, MAPK15 and SCRIB form a plausible core network influencing cholesterol deposition in leg muscle.

In summary, this work provides a genomic resource and population genetics framework for Meigu Yanying chickens and highlights candidate regions and genes related to growth and meat quality. These results lay a foundation for genomic selection, marker-assisted breeding and genetic resource management in this local breed, and offer molecular targets for developing Liangshan chicken lines combining high productivity, desirable meat quality and improved nutritional attributes. To better evaluate the breeding utility of the detected signals, larger datasets with more controlled/replicated phenotyping will be needed to estimate trait heritability and quantify the proportion of phenotypic variance explained by associated loci. Future studies will incorporate more detailed nutritional indices (e.g., amino acid composition and tryptophan/hydroxyproline ratio), controlled stress challenge designs, and comparisons with commercial broiler lines/crosses to further dissect pathways underlying muscle protein deposition and adaptation.

## Figures and Tables

**Table 1:** Descriptive statistics of four traits in Meigu Yanying chickens (n = 211).

Traits	Num.	Mean	SD	Max.	Min.	CV (%)
LMW	211	244.49	65.41	421.47	104.74	26.75
LW	211	85.31	20.83	158.21	46.91	24.42
LMTP	211	3.32	1.11	6.16	1.26	33.47
LMTC	211	0.08	0.05	0.22	0.00	57.83

Note: LMW, leg muscle weight (g); LW, liver weight (g); LMTP, leg muscle total protein concentration in leg muscle extract (g/L); LMTC, leg muscle total cholesterol normalized to total protein (mmol/g protein). Values are presented as mean, standard deviation (SD), and minimum and maximum. The coefficient of variation (CV, %) was calculated as CV = (SD/mean) × 100.

**Table 2:** Summary of genome-wide association results for four traits in Meigu Yanying chickens.

Trait	Lead SNPs	Sig. SNPs ^1^	Sug. SNPs ^2^	Top SNP	*p*_Best	Genes ^3^
LMW	57	1	17	5_55268309_C_T	2.89 × 10^−8^	139
LW	62	5	22	2_86456028_G_A	1.37 × 10^−8^	140
LMTP	37	3	3	3_82338258_G_A	4.56 × 10^−8^	74
LMTC	89	9	28	1_139186656_C_T	5.00 × 10^−9^	174

Note: ^1^ Genome-wide significant SNPs (*p* < 0.05/M); ^2^ Suggestive SNPs (1/M ≤ *p* < 0.05/M); ^3^ Number of candidate genes within ±100 kb of lead SNPs.

**Table 3:** Summary of GO enrichment analysis for the four traits.

Trait	Top SNP	*p*_Best	No. of GO Terms	Representative GO Term ^1^
LMW	5_55268309_C_T	2.89 × 10^−8^	28	GO:0009987
LW	2_86456028_G_A	1.37 × 10^−8^	6	GO:0008150
LMTP	3_82338258_G_A	4.56 × 10^−8^	6	GO:0048518
LMTC	1_139186656_C_T	5.00 × 10^−9^	73	GO:0009987

Note: ^1^ The representative GO term is the GO biological process with the lowest q value for that trait and is used to summarize the main direction of functional enrichment.

## Data Availability

The data presented in this study are available on request from the corresponding authors.

## Supplementary Materials

The following supporting information can be downloaded at: https://www.mdpi.com/article/10.3390/ani16040540/s1, Table S1: Table_S1_Meigu_Yanying_GWAS_GO.xlsx.
